# The Effects of Personalized Nudges on Cognitively Disengaged Student Behavior in Low-Stakes Assessments

**DOI:** 10.3390/jintelligence11110204

**Published:** 2023-10-28

**Authors:** Burcu Arslan, Bridgid Finn

**Affiliations:** 1 Educational Testing Service Global, Strawinskylaan 929, 1077 XX Amsterdam, The Netherlands; 2 Educational Testing Service, Princeton, NJ 08544, USA; bridgid.finn@fmr.com

**Keywords:** test-taking behavior, problem-solving, engagement, metacognition, effort monitoring, nudges, low-stake assessments

## Abstract

In educational settings, students rely on metacognitive processes to determine whether or not to exert effort. We investigated ways to minimize cognitively disengaged responses (i.e., *not-fully-effortful* responses) during a low-stakes mathematics assessment. Initially, we established theory-driven time thresholds for each item to detect such responses. We then administered the test to 800 eighth-graders across three conditions: (a) control (*n* = 271); (b) instruction (*n* = 267); and (c) nudge (*n* = 262). In the instruction condition, students were told to exert their best effort before starting the assessment. In the nudge condition, students were prompted to give their best effort following each first-attempt response that was both incorrect and not-fully-effortful. Therefore, students had multiple opportunities to adjust their level of effort. Nudges, but not effort instruction, significantly reduced students’ not-fully-effortful responses. Neither the nudges nor the effort instruction significantly impacted performance. In a post-test survey, most students reported that they received nudges whenever they did not know the answer (55%). Overall, these findings suggest that while nudges reduce cognitively disengaged responses, most students appear to strategically modulate their level of effort based on self-monitoring their knowledge and response effort.

## 1. Introduction

When designed and used appropriately, assessments support learning (e.g., see Bennett 2011 for a discussion of the benefit of formative assessments *for* learning, or see Rowland 2014 for the role of the testing effect in supporting learning) and provide stakeholders such as students, teachers, and policy makers with information about student learning outcomes (i.e., what students know and can do). During problem-solving, cognitively engaged students go through several cognitive processes, such as encoding the presented item through perception and working memory, problem solving, and/or reasoning through activating their declarative and procedural knowledge. In addition to the cognitive processes, they also go through metacognitive processes, such as monitoring and controlling their own cognitive processes, knowledge, and skills (e.g., see Anderson and Fincham 2014; Brown 1987; Schneider and Artelt 2010). Students might also rely on these metacognitive processes to determine whether they will cognitively engage with the problem or not (i.e., to give effort or not; for example, see Efklides 2011; Miele and Scholer 2018). Similarly, in the educational assessment context, when students are presented with an item, based on their initial encoding of the screen and/or item stem, they might quickly evaluate their declarative and procedural knowledge about the presented item, and estimate the difficulty of the item and the amount of effort they will need to give to provide a response. Based on their initial, swift evaluation of their familiarity with the question or *feeling of knowing* (Reder and Ritter 1992; Paynter et al. 2009), they might make a judgment to go through the solution process, skip the item (if it is possible), or provide an answer without going through solution processes (i.e., responding without giving their best effort).

When the assessment task is high stakes for the student (i.e., test performance has formal consequences for the student), students are more motivated to perform well than when the assessment task is low stakes (i.e., test performance has no formal consequences). When motivated, and when the student judges that they have content knowledge relevant to answering the question, they will be more likely to cognitively engage with the task by expending effort to solve the problem due to having motivation to perform well (see Finn 2015 for measuring motivation in low-stake assessments). However, when the assessment is low stakes for the student (e.g., large-scale national or international assessments, such as The National Assessment of Educational Progress (NAEP), or The Programme for International Student Assessment (PISA)), motivation to cognitively engage with the task and provide effortful responses are reduced (Finn 2015; Gneezy et al. 2019; Wise 2017). It is important to detect cognitively disengaged student behavior because, both in low stakes and high stakes assessment contexts, the inferences about students’ knowledge, skills, and abilities are made with the assumption that they gave their best effort when taking the test.

In the multiple-choice assessment context, one of the well-established indicators of cognitive disengagement is called rapid guessing behavior ((RGB) Goldhammer et al. 2017; Wise and Kong 2005; see Wise 2017 for a review; see also Nagy et al. 2023 for detecting disengagement based on performance decline). RGB is defined as responses that have an unrealistically short response time, which indicates that examinees did not plausibly complete all required (meta)cognitive processes and suggests that they did not provide a response effortfully. The Demands-Capacity Model ((DCM) Wise and Smith 2011; see also Wise 2017) provides a theoretical explanation for RGB. According to the DCM, when an item is presented, the student first evaluates their effort capacity against the resource requirements of the item. The student cognitively engages in solving the task if they judge that their effort capacity is adequate; if not, they show RGB. As both the difficulty of the item and the effort capacity of the student can fluctuate across different items, the occurrence of RGB during a test can fluctuate (see also Goldhammer et al. 2017 for the factors that affect test-taking engagement). The existence of these two distinct response behaviors (i.e., RGB and solution behavior) is supported by three different types of evidence: (a) significant difference in time spent on item; (b) significant difference in accuracy, and (c) the amount of psychometric information between RGB and solution behavior (Wise 2017). Wise (2017) discusses these two distinct response behaviors within the Dual-Process Theory which explains human decision-making in terms of two cognitive systems, namely System 1 and System 2 (Kahneman 2011; Stanovich et al. 2000). Wise (2017) conceptualized RGB as a manifestation of System 1, which is quick, intuitive, and requires little cognitive effort, and solution behavior as a manifestation of System 2, which is slower, more reflective, cognitively demanding, and requires analytical processing.

What are the methods to detect and classify whether a response is based on RGB or solution behavior? Several methods have been proposed (see Wise 2017 for a review; also see Wise 2019 for a more recent method). A common procedure in data-driven methods is, after the assessment data are collected, to set an item time-based threshold (i.e., based on the response time), which reflects an unrealistically quick response. The responses below the item threshold are classified as RGB, and the responses above the threshold are classified as solution behavior. While all responses classified as RGB are expected to be not effortful, *not all* responses classified as solution behavior are expected to be effortful, which might indicate partial engagement (Lindner et al. 2019; Wise and Kuhfeld 2021). One of the commonly used methods, due to its simplicity, is called the normative threshold method (Wise and Ma 2012; see also Soland et al. 2021 for a comparison of different threshold setting methods). In the normative threshold method, the time threshold for an item is set at a certain percentage of the average time that the examinee spent on the item (e.g., 10% of the mean item time with a maximum threshold value of 10 s; Wise and Ma 2012; see also Bulut et al. 2023 for a method to find the optimal threshold). More recently, recognizing that the RGB measure can be too conservative, the time threshold suggestions are extended from 10% to 20% or 30% of the average item time (Wise and Kuhfeld 2020, 2021). For example, if the average time students spent on the item was 80 s, the normative threshold method of 10% would be 8 s to detect RGB, and the normative threshold method of 20% and of 30% would be 16 and 24 s, respectively. After setting thresholds for each item and making classifications between RGB and solution behavior, *response time effort* ((RTE) Wise and Kong 2005) is calculated. RTE is the proportion of an examinee’s responses that were classified as solution behaviors. RTE is used as a continuous index of students’ overall level of engagement during the assessment task (see Wise and Gao 2017 for a more generalized index for effort). In general, these data-driven methods are applied to detect, report, or statistically manage cognitively disengaged behavior *after* the assessment task is complete (e.g., for a comparison of different treatments, see Deribo et al. 2023).

Is it possible to intervene on cognitively disengaged student behavior *during* the assessment task so that students can shift their behavior from System-1-driven RGB to System-2-driven solution behavior? Research in behavioral and decision sciences has started to develop System-1-related intervention strategies through *nudges* (for a meta-analysis, see Mertens et al. 2022; see also Hertwig and Grüne-Yanoff 2017 for nudges vs. boosts) that are grounded in dual-process theories of cognition (Evans 2008; Kahneman 2011). These approaches recognize that decision making is not always rational (Simon 1955), but often relies on simpler and less computation-heavy mental processes due to constraints of time, complexity of the presented information, and computational power (see also Lieder and Griffiths 2020 for resource-rational analysis). Nudge interventions aim to change behavior by restructuring the environment that the behaviors are occurring in (i.e., altering the *choice architecture*, see Münscher et al. 2016 for a review of choice architecture techniques) by providing a choice to engage in the suggested behavior or not (Thaler and Sunstein 2008; Thaler et al. 2013). Subtle changes in the environment can have a major impact. For example, changing the default to *opt-in* for organ donation when people obtain their drivers’ licenses was shown to greatly increase the number of voluntary donors. The rationale for this change was that it took more effort to opt-out than to stick with the opt-in default, which greatly increased the numbers (Thaler and Sunstein 2008). Thaler et al. (2013) also discuss warnings as an effective method to adaptively change behavior, with the caveat that effectiveness declines if the warnings occur too frequently. A meta-analysis of nudges has shown that there is a fairly substantial effect of nudges (Mertens et al. 2022), although follow up research suggests that the effect is eliminated after adjusting for publication bias (see Maier et al. 2022). Nudging is not widely used in testing contexts; however, nudging in the educational context was found to show positive changes in some students’ behavior, under specific conditions (e.g., van der Sande et al. 2023; Levitt et al. 2016; see also Damgaard and Nielsen 2018; and Weijers et al. 2021, for a theory and guidelines for using nudges in educational contexts).

As a first study, Wise et al. (2006) conducted a controlled experiment with university students in which RGB was detected *during* the assessment and students were provided with warnings (i.e., “effort-monitoring”; see also Wise et al. 2019 for sending warnings to proctors). These warnings were similar to the nudges in a way, in that the goal was to change behavior without restricting students’ freedom of choice but being more explicit than the nudges under the choice architecture. The overall goal of the warnings was to prevent continued RGB behavior. Students were presented with a maximum of two warnings, which were introduced only after students had demonstrated RGB on three consecutive items. The first warning text was as follows: “*Your responses to this test indicate that you are not giving your best effort. It is very important that you try to do your best on the tests you take on Assessment Day. These assessment data are used by the university to better understand what students learn at <the university>, and what improvements need to be made. In addition, <the university’s> assessment data are reported to the state as evidence of what <the university’s> students know and can do.*” (Wise et al. 2006, p. 24). The second warning text was as follows: *“<Examinee Name>, your responses continue to indicate that you are not trying to do your best on this test. These tests are very important, and you need to give them serious attention. Students who do not take Assessment Day activities seriously may be required to attend an Assessment Day make-up session.”* (Wise et al. 2006, p. 24). Wise et al. showed that presenting these warnings significantly increased university students’ response time effort (RTE) but not their overall test score, although there was a positive shift compared to the control condition (i.e., no warning). The authors pointed out future directions to investigate whether warnings can be effective for the K-12 population or in environments like NAEP, and whether the effort-monitoring approach can be expanded beyond multiple-choice items.

### The Present Study

To the best of our knowledge, we are the first to investigate the effects of *nudging* and *effort instruction* designed to encourage 8th-grade students to give their best effort during a NAEP-like mathematics assessment. Our study examines the effects of these manipulations on cognitively disengaged student behavior, performance, as well as post-test survey measures (i.e., perceived difficulty, expected performance, and feeling very nervous). Although there are several similarities with the present study and Wise et al.’s (2006) study, there are also several major differences between the two. For a comparison, see Table 1.

The first difference between Wise et al. (2006) and our study is related to the measure we used to detect cognitively disengaged student behavior. While Wise et al. used RGB as a measure, we used *not-fully-effortful* responses. Unlike RGB, which is defined as unrealistically quick responses, we define not-fully-effortful responses as responses that are below the solution time threshold of an extremely proficient student who can read really fast according to the population and has all the knowledge, skills, and efficient strategies to respond correctly to an item with a very quick retrieval from memory (i.e., 50 ms). When items are not text heavy and do not require extensive cognitive processes, items deemed not-fully-effortful response and those deemed as RGB, theoretically, can overlap.

The second difference is based on the experimental conditions. While Wise et al. (2006) compared only a warning condition to no warning condition, in the present study, we compared a nudge condition to an effort instruction condition, in addition to comparing it to a control condition.

The third difference is the method to detect cognitively disengaged student behavior. As we discussed above, most of the existing methods to set item time thresholds require initial data about item time. In our study, we did not have previous data about the item response times. Therefore, we set item time thresholds based on a theoretical, cognitive modeling method (Arslan et al. 2021). Although it is out of the scope of this paper, in a nutshell, the theory behind the method is based on a cognitive architecture called Adaptive Control of Thought-Rational ((ACT-R) Anderson 2007). ACT-R is a computer implementation of a unified theory of cognition. That is, ACT-R theory includes the fixed mechanisms and structures that underlie human cognition, and it can simulate human cognition and behavior together with their timing. ACT-R has been used in different fields spanning from cognitive science, developmental psychology, human–computer interaction, and education (e.g., intelligent tutoring systems such as Cognitive Tutor^®^) to predict and explain learner behavior (for a list of publications, see http://act-r.psy.cmu.edu/publication/, accessed on 30 June 2023). ACT-R has default parameters for timing of perception, action, and cognitive processes based on empirical research. For example, it takes 50 ms to encode a visual chunk of information, 200 ms to create a working memory chunk, and 250 ms for motor movement preparation after deciding to press a button.

**Figure A1:**
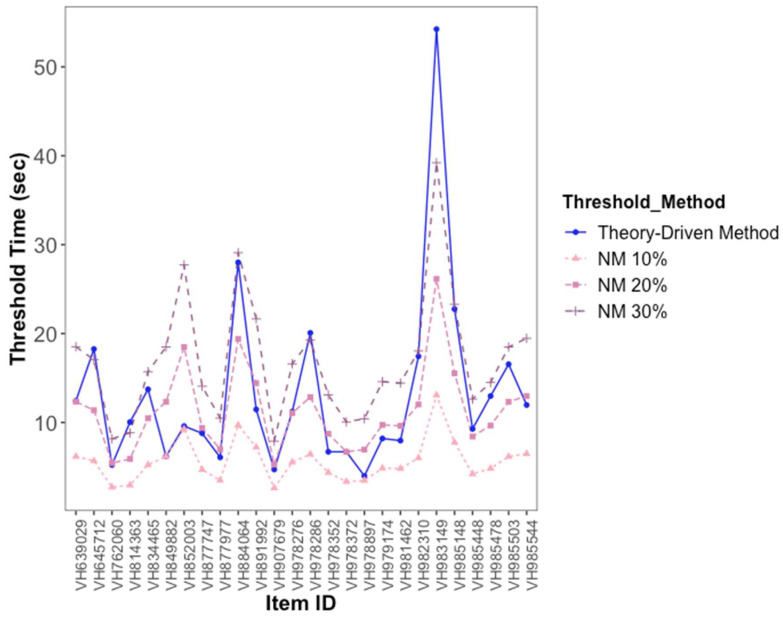
A comparison of item time thresholds between the theory-driven method that we used in this study and the normative threshold method (NM) with different percentages (10%, 20%, and 30%).

The procedure of calculating the item time thresholds for detecting not-fully-effortful responses requires calculating the minimum time needed for completing each information processing step. To do so, the theory-driven method requires: (a) counting the total number of words in an item (if any), (b) picking a very fast reading rate (i.e., considering the population) based on the prior literature (e.g., Carver 1992), (c) multiplying the values in steps *a* and *b*, (d) with the help of a domain expert, performing a cognitive task analysis to identify mental processes of a very proficient student who has all the knowledge, skills, and efficient strategies to respond correctly to an item with a very quick retrieval from memory, (e) using ACT-R’s micro-level timing values to calculate the minimum time required to perform the identified mental processes, (f) calculating the minimum time for response actions (e.g., mouse clicks) by leveraging previous human–computer interaction literature (e.g., Ramkumar et al. 2017), (g) adding the values calculated in steps *c*, *e*, and *f* to calculate the minimum required time for responding to an item in a cognitively engaged way (i.e., a very conservative, quickest possible solution time). The responses below the defined item time thresholds are classified as not-fully-effortful responses and above the threshold as solution behavior. Note that the solution behavior does not necessarily indicate that all responses are fully effortful. See Figure A1 in Appendix A for the comparison of item thresholds between the normative threshold method (with 10%, 20%, and 30% of the mean item time) and the theory-driven method that we applied in the present study.

A fourth difference between Wise et al. (2006) and our study is related to item navigation. In Wise et al., university students had to answer each item and they were not allowed to go back to an item after they had submitted their answer. In the present study, similar to NAEP, students could omit answers and they were able to navigate between items within a block.

Finally, the warning and nudge algorithms were different between the two studies. Wise et al.’s study introduced at most two warnings. The first warning was presented after detecting three consecutive RGBs. The second warning was presented if the examinees had another three consecutive RGBs. On the other hand, in the present study, students were presented with a nudge to give their best effort following their each first-attempt response that was both incorrect and not-fully-effortful (i.e., if a student visited an item multiple times without a response or went back to an item that they previously provided a response and quickly changed their answer, they were *not* presented with a nudge). Therefore, students had multiple occasions to adjust their effort both for the last item with which they engaged and for the remaining items. In the current study, we investigated four research questions:

1. Are there any significant differences in item response disengagement in the nudge condition compared to the control condition and effort instruction conditions?

2. Are there any significant differences in performance (i.e., item score) in the nudge condition compared to the control condition and effort instruction conditions?

3. How do students behave when presented with a nudge screen (i.e., do they indicate that they gave their best effort, do they go back to the item, do they interact with the response field when they go back to the item)?

4. Are there any significant effects of nudges on students’ metacognitive measures (i.e., perceived effort, perceived difficulty, expected performance, and feeling very nervous) compared to the control and effort instruction conditions?

Considering Wise et al.’s (2006) study, we hypothesized that introducing nudges would significantly decrease cognitively disengaged responses. Moreover, Wise et al. found a positive shift in the test performance in their experimental condition (warning condition) compared to the control condition. Similarly, we also expected a positive shift in performance in the nudge condition compared to the control condition. The remainder of the analyses reported in the manuscript were exploratory. To preview, the results showed that nudging students significantly reduced not-fully-effortful responses compared to control and effort instruction conditions. Nudges had no significant impact on performance compared to control and effort instruction conditions. In a post-test survey, most students agreed that they received nudges whenever they did not give their best effort (67%) and whenever they did not know the answer (55%). Although there were trends in the data, there were no significant differences in metacognitive measures in post-test survey (i.e., perceived effort, perceived difficulty, expected performance, and feeling very nervous) between the nudge and control conditions, and the nudge and effort instruction conditions. Overall, RTE had a positive significant association with the ratings for perceived effort and did not have a significant association on feeling very nervous. Moreover, the actual math score had a negative significant association on the ratings for perceived difficulty and had a positive significant association on expected performance.

## 2. Methods

### 2.1. Participants

We collected data from 800 8th-grade students (*Gender*: 47.2% Female, 0.3% Other; *Race*: 85% White, 8.1% Hispanic/Latino, 3.6% Black/African American, 3.3% Other; *Social Economic Status (i.e., whether the student participated in the free or reduced lunch program)* = 57.8% Yes; *Accommodations (i.e., the student receive accommodations on Title I assessments required by No Child Left Behind)* = 7.5% Yes, 11.1% NA; *Current mathematics grade*: 30% A, 22.6% B, 18.8% C, 12.6% D, 13.8% F, 2.2% NA) from 8 different schools from 7 different states in the U.S.

### 2.2. Materials

The assessment consisted of two mathematics blocks. Each block had 15 items, which included the same items and block design that were used in Lehman et al. (2022). The items covered different mathematical topics, such as knowing and applying the properties of integer exponents to generate equivalent, numerical expressions, and understanding and applying the Pythagorean Theorem. The assessment included a variety of item formats. More specifically, ten single-select multiple-choice, six numeric-entry, four multiple-select multiple-choice, two drag-and-drop, two inline-choice, one grid, one composite (two numeric entry and one inline-choice parts), one zone, one point-and-click graph, one equation editor, and one graph items. We excluded four items from the analyses since the item formats could not be machine scored during assessment (i.e., one zone, one point-and-click graph, one equation editor, and one graph item). Thus, the analyses include 26 items.

### 2.3. Design and Procedure

In a between-subject design, students were randomly assigned to one of the following three conditions: (a) control (*n* = 271); (b) effort instruction (*n* = 267); and (c) nudge (*n* = 262). Because the assessment delivery platform we used cannot randomly assign items into the forms, the total of 30 items are first divided into 6 sets (i.e., 5 items each). These six sets were divided between two blocks (i.e., three sets in each block). Subsequently, block positions and set positions within a block were randomized, resulting in six forms. Finally, these six forms were assigned to each of the three conditions. Therefore, in total, the study included 18 forms.

We created unique IDs for each student in a classroom and randomly assigned each test form to a unique ID so that they could not be identified. The assessment platform link was shared with the teachers and students logged in to the platform with their unique IDs. All students completed the test within one-classroom period as a part of a grade 8 mathematics class. After completing the mathematics assessment, students were presented with three sequentially presented post-test screens that included 6-point Likert scale questions asking about students’ perceived effort and difficulty, expected performance, whether they felt nervous (in all conditions), and their experience about the nudges (only in the nudge condition). Since it was a classroom activity, students did not receive any incentives. However, the schools received $20 per student who completed the assessment.

#### Experimental Conditions

*Control Condition*: The items were presented with a NAEP-like interface such that students could freely navigate between items. Students were allowed to navigate between items by clicking the tabs within a block. They could omit answers (i.e., skip items) or change their answers during a revisit. Before starting to work on the mathematics assessment, students were presented with a tutorial screen, which included a video explaining how to navigate the assessment and use the available tools. At the end of each block, there was a review screen in which students could see the items that they had not answered and could navigate back to those items. Once students moved from one block to another, they were not allowed to go back to the previous block.

*Instruction Condition*: This condition was the same as the control condition, except the fact that students were presented with a screen after the tutorial indicating that “*Please note that in addtion to your final response to each item, YOUR INTERACTIONS with each item will also be evaluated. It is very important that you give your best effort and try to do your best. These assessment data are used to better understand what students know and can do.*” At the end of this effort instruction, students were required to click the check box “*I understand that my interactions with each item will be evaluated, and I need to give my best effort and try my best*” before they started the mathematics task.

**Figure A2:**
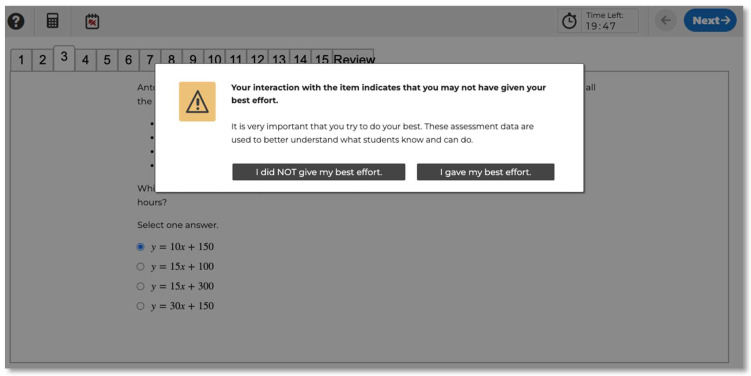
A screenshot of the first nudge screen.

**Figure A3:**
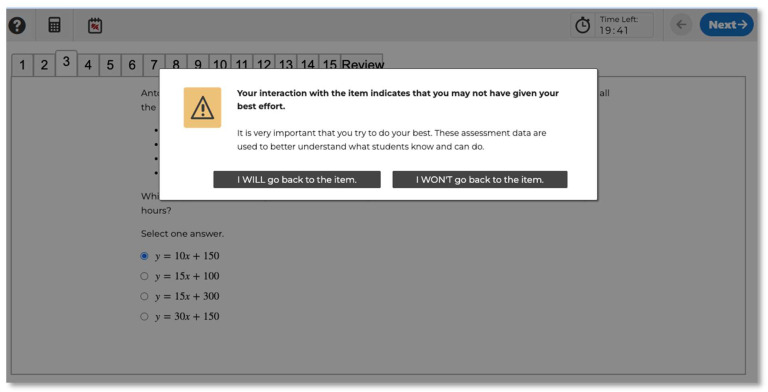
A screenshot of the follow-up nudge screen which was presented after students clicked on one of the buttons on Figure A2.

*Nudge Condition*: Except for the fact that students were presented with a nudge to give their best effort following each first-attempt response that was both incorrect and not-fully-effortful, the nudge condition was the same as the control condition. As we discussed in the introduction, using a theory-driven method, we initially identified a very fast solution time threshold for each item, indicating an estimate of the minimum time required for a very proficient, cognitively engaged student to solve an item (Range: 3.985–54.241 s; *M* = 13.924; *SD* = 10.157; Mdn = 11.343). If a student’s response was incorrect and below the predefined item time threshold, they were presented with a nudge. However, if a student visited an item multiple times without a response or went back to an item that they previously provided a response and quickly changed their answer, they were *not* presented with a nudge. Thus, nudges were presented only during students’ first visit in which they provided a response (i.e., first response). The nudge pop-up screen included a similar text with the effort instruction condition: “*Your interaction with the item indicates that you may not have given your best effort. It is very important that you give your best effort and try to do your best. These assessment data are used to better understand what students know and can do*”. Below this text, students were required to select one of the two buttons indicating either “*I did NOT give my best effort*” or “*I gave my best effort*”. After they clicked one of the two buttons, they had to indicate whether they wanted to go back to the item or move to the next item by clicking one of the two buttons presented at the same location with the previous buttons “*I WILL go back to the item*” and “*I WON’T go back to the item*” independent of their selection related to their effort (see Figure A2 and Figure A3 in Appendix B for screenshots of the nudge screens).

### 2.4. Analyses

In this subsection, we first report our data preprocessing and analysis approach. Subsequently, we report the features we created and specifics of the analyses, and the models for each research question we outlined in the Introduction.

#### 2.4.1. Data Preprocessing and Analysis Approach

The initial parsing of the JavaScript Object Notation (JSON) files for the process data, most feature creation, and a subset of merging different files (i.e., log data, response data, and background information data) were conducted using NumPy (Harris et al. 2020) and Pandas (McKinney 2010) in Python 3 (Van Rossum and Drake 2009). Subsequently, cocron (Diedenhofen 2016), tidyverse (2.0.0; Wickham et al. 2019), lme4 (1.1.32; Bates et al. 2015), emmeans (1.8.5; Lenth 2023), and sjPlot (2.8.14; Lüdecke 2023) R programming language packages (R Development Core Team 2023) were used to conduct a significant test for reliability coefficients, create new features, conduct analyses, obtain estimated marginal means, and create figures, respectively.

All analyses were conducted on a subset of the data which included the first attempted responses. The reason for this choice was related to the fact that nudges were presented only after students’ first-attempt responses. Therefore, omitted responses were not included in the analyses.

To calculate students’ response time effort scores (RTE) for each student, the number of items that are classified as solution behavior was divided by the total number of items that had a response. Therefore, the range for the RTE score was between [0,1].

Unless otherwise stated in the subsections below, we conducted generalized mixed effect models. All models included condition, block, and current math grade as fixed effect factors. As random factors, unless otherwise stated, the models included random intercepts for items and students nested under teachers. The models did not include random slopes for block due to singular fit. Whether to include interactions between fixed effect factors were exploratory and decided via model comparisons using the Akaike Information Criterion (AIC), which takes into account both the goodness of fit and the simplicity of the model. We use ∆AIC to report the difference between the AIC score of the previous model and the next model (i.e., AIC_previous model_ − AIC_next model_), and selected the next model if AIC was greater than the rule of thumb number of 2 (Burnham and Anderson 2002). We ran the models with *bobyqa* optimizer. If a model did not converge, we tried different optimizers (i.e., Nelder_Mead, nloptwrap). If the model still did not converge, we selected a simpler model.

#### 2.4.2. Response Disengagement

To investigate RQ1, we conducted analyses by using item response disengagement (i.e., whether a response is not-fully-effortful) as a dependent variable and compared the nudge condition with the effort instruction and control condition.

In all conditions, item response disengagement included the factor “Not Fully Effortful” if the response was both incorrect and below the theoretical item time threshold; otherwise, the factor “Effortful” indicating solution behavior. Note that all responses classified as “Not Fully Effortful” are expected to be not fully effortful; however, *not* all responses classified as “Effortful” are expected to be effortful.

An initial generalized mixed-effects model with binomial family (i.e., condition, block, and current math grade as fixed effect with random intercepts with items and students nested under teacher) was compared with a model that included a two-way interaction between condition and block. The initial model was a better fit to the data (∆AIC = −3.793). Subsequently, we also added a two-way interaction between condition and current math grade and compared with the second model. The second model was a better fit to the data (∆AIC = −4.017). Therefore, the final model (i.e., initial model) included condition, block, and current math grade as fixed effects without any interaction term as well as random intercepts for items and students nested under teacher.

#### 2.4.3. Performance

To investigate RQ2, we conducted analyses with item final score as a dependent variable and compared the nudge condition with the effort instruction and control conditions. The items that required a single response were scored as 0 and 1 (i.e., single select multiple choice, numeric entry with a single entry field). The rest of the items scored between 0 and 1 based on the proportion of the correct options (e.g., students are scored as 0.5 if one of the two response fields was correct). Similar to the item response disengagement variable, the analyses included a subset of the data for the first-attempt responses, meaning that the omitted responses were not included in the analyses.

Initial generalized mixed-effects model with binomial family was compared with a model that included a two-way interaction between condition and block. The initial model was a better fit to the data (∆AIC = −0.540). Subsequently, we also added a two-way interaction between condition and current math grade and compared with the initial model. The initial model was a better fit to the data (∆AIC = −13.580). Therefore, the initial model without interactions was selected as the final model (i.e., condition, block, and current math grade as fixed effect with random intercepts for items and students nested under teacher).

#### 2.4.4. Students Behavior after Receiving a Nudge

We investigated RQ3 by reporting the descriptive statistics related to nudge conditions. More specifically, we report the mean, standard deviation, maximum and minimum number of (a) nudges that each student received as a result of their detected cognitively disengaged behavior; (b) selection of each student’s “*I did NOT give my best effort*” and “*I gave my best effort*”; (c) selection of “*I will go back to the item*” and “*I will NOT go back to the item*”. Also, we present overall percentages of these variables based on the total number of observations.

#### 2.4.5. Metacognitive Measures

To investigate RQ4, we conducted analyses with students’ responses to the 6-point Likert scale post-test questions as dependent variables (i.e., 1 = Strongly Disagree, 2 = Disagree, 3 = Somewhat Disagree, 4 = Somewhat Agree, 5 = Agree, 6 = Strongly Agree). The self-reported measures included questions about students’ perceived effort, perceived difficulty, expected performance, and how nervous the mathematics activity made them.

We followed the tradition that ordinal variables with five or more categories can be used as continuous (Johnson and Creech 1983; Norman 2010; Sullivan and Artino 2013; Zumbo and Zimmerman 1993) and used linear regression. Since the linear mixed-effect models did not converge even when the models included only random intercepts for students, we fitted linear regression models (except to investigate expected performance for which we fitted a logistic regression model) for each self-report measure after excluding students who did not have any detected cognitively disengaged behavior (i.e., RTE = 1). To investigate perceived effort, we compared a model that included condition and RTE with a model that included the interaction between the two. The model without interaction was a better fit to the data (∆AIC = −2.833). To investigate perceived difficulty, we compared a model that included condition and total proportion correct with a model that included the interaction between the two. The model without interaction was a better fit to the data (∆AIC = −3.135). To investigate expected performance, we compared a model that included condition and total proportion correct with a model that included the interaction between the two. The model without interaction was a better fit to the data (∆AIC = −1.991). Finally, to investigate how nervous the mathematics activity made them in the nudge condition compared to effort instruction and control conditions, we compared a model that included condition and RTE with a model that included the interaction between the two. The model without the interaction was a better fit to the data (∆AIC = −0.831).

## 3. Results

Although students did not have to respond any of the items, the proportion of responded items was high (Block 1: *M* = 0.932; *SD* = 0.150; Block 2: *M* = 0.866; *SD* = 0.151). The reliabilities (coefficient alphas) of the mathematics test were 0.82 (0.80 after removing not-fully-effortful responses) for the control condition, 0.80 (0.76 after removing not-fully-effortful responses) for the effort instruction condition, and 0.84 (0.83 after removing not-fully-effortful responses) for the nudge condition. While introducing nudges did not significantly increased the reliability compared to control condition (Nudge-Control: *F* = 1.156, *df1* = 261, *df2* = 269, *p* = .238), it significantly increased the reliability compared to effort instruction, these trends were not statistically significant; Nudge-Instruction: *F* = 1.376, *df1* = 261, *df2* = 266, *p* = .009).

In the next subsections, we present the results of our analyses for the research questions related to item response disengagement (RQ1), item score (RQ2), students’ test-taking behavior after receiving a nudge (RQ3), and self-report measures (RQ4; i.e., perceived difficulty, perceived effort, expected performance, how nervous the mathematics activity made them).

### 3.1. Response Disengagement

Overall, students had a higher response time effort RTEs in both blocks in the nudge condition (M_Block 1_ = 0.945, *SD* = 0.104; M_Block 2_ = 0.888, *SD* = 0.180) compared to the control condition (M_Block 1_ = 0.931, *SD* = 0.136; M_Block 2_ = 0.857, *SD* = 0.196) and the effort instruction condition (M_Block 1_ = 0.919, *SD* = 0.155; M_Block 2_ = 0.854, *SD* = 0.213).

As we mentioned in the Analysis subsection above, the final model for item response disengagement included condition, current math grade, block as fixed effects, and random intercepts for items and students nested under teachers. Table 2 shows the odds ratio, confidence intervals, and *p*-values of the final model for item response disengagement (see Table A1 in Appendix C for the estimates, standard errors, *z*-values and *p*-values of the final generalized mixed-effects model).

Confirming our hypothesis, nudging students significantly decreased the cognitively disengaged students compared to control condition (i.e., the odds ratios of cognitively disengaged item response was significantly higher in the control condition compared to the nudge condition). A similar effect was observed in the nudge condition compared to the effort instruction condition^1^.

### 3.2. Performance

**Figure 1:**
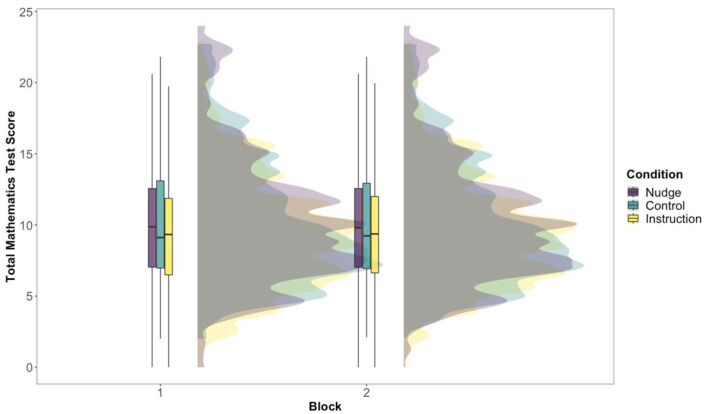
Distribution of total mathematics test score by condition and block.

Similar to Wise et al.’s (2006) study, there was a positive shift in students’ overall mathematics test scores (see Figure 1) in the nudge condition (M_Block 1_ = 10.297, *SD* = 4.550, Mdn_Block 1_ = 9.87; M_Block 2_ = 10.296, *SD* = 4.535, Mdn_Block 2_ = 9.80) compared to the control condition (M_Block 1_ = 10.165, *SD* = 4.358, Mdn_Block 1 _ = 9.11; M_Block 2_ = 10.104, *SD* = 4.283, Mdn_Block 2_ = 9.23). The same pattern did not hold for the effort instruction condition (M_Block 1_ = 9.576, *SD* = 4.161, Mdn_Block 1_ = 9.33; M_Block 2_ = 9.756, SD = 4.094, Mdn_Block 2_ = 9.37).

As we mentioned in the Analysis subsection above, the final model for item score included current math grade, condition, and block as fixed effects without any interactions, and random intercepts for items and students nested under teachers. Table 2 shows the odds ratios, confidence intervals, and *p*-values of the final generalized mixed-effects model (see Table A1 in Appendix C for the estimates, standard errors, *z*-values*,* and *p*-values of the final generalized mixed-effects model for item score).

The results showed that: (a) there was no significant difference between the nudge condition and the control conditions, and the nudge and effort instruction conditions, (b) students performed significantly lower in the second block, and (c) showing external validation of our mathematics test, students whose math grades were lower than A in their current math class (i.e., B, C, D, F) performed significantly lower in our mathematics test compared to the students whose grades were A.

### 3.3. Student Behavior after Receiving a Nudge

**Figure 2:**
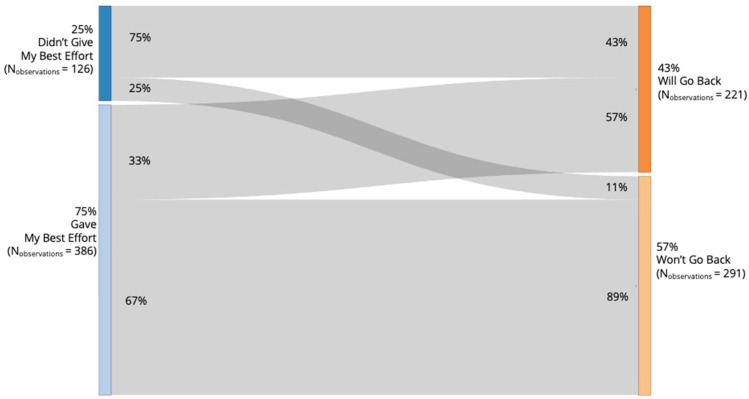
A Sankey diagram showing the number and percentage of students’ selections among overall observations.

As we mentioned in the Methods section, students were presented with a nudge screen in which they were required to select one of the two buttons that included a statement related to their effort (i.e., “I gave my best effort” and “I did NOT give my best effort”). Subsequently, independent of their selection, they were required to select one of the two buttons that included a statement about their choice about their next step (i.e., “I WILL go back to the item” and “I WON’T go back to the item”). In total, 142 students who were in the nudge condition (54%) received at least one nudge (M = 3.69; SD = 3.65, Max = 17, Min = 1). Among these students, 71 students (50%) selected “I did NOT give my best effort” at least once (N_observations_ = 126; M = 1.77; SD = 1.77; Max = 14; Min = 1) and 114 students (80%) selected “I will go back to the item” at least once (N_observations_ = 221; M = 1.94; SD = 1.61; Max = 9; Min = 1) regardless of their selection about their effort. Figure 2 presents a Sankey diagram showing the number and percentage of students’ selections among overall observations (N = 512).

Among the 114 students who went back to items (N_observations_ = 221), 84 students (74%) interacted with the response field (i.e., they changed their answer, or they kept their previous answer after initially changing their answer) at least in one item that they received a nudge (N_observations_ = 115; M = 1.37; SD = 0.71; Max = 5; Min = 1), and 26 students (23%) left the item without any interaction at least once (N_observations_ = 44; M = 1.69; SD = 1.41; Max = 7; Min = 1). Note that due to the technical problems in the data, we do not have information about students’ behavior for 62 observations (28%).

**Figure 3:**
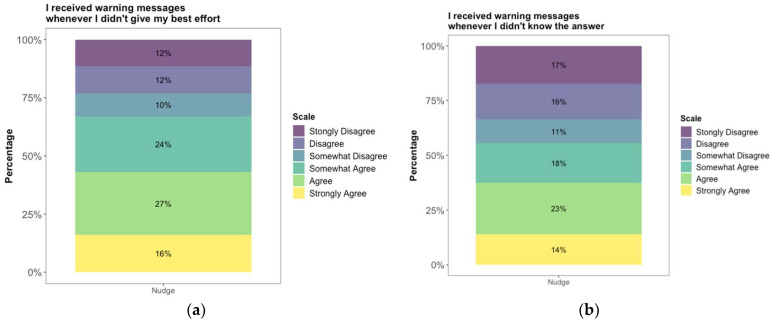
Percentage of students’ ratings in the nudge condition for the statements: (**a**) “I received warning messages whenever I did not give my best effort”, and (**b**) “I received warning messages whenever I did not know the answer”.

In the post-test survey, we also asked students to rate their experience based on the following two statements: (a) “I received warning messages whenever I did not give my best effort”, and (b) “I received warning messages whenever I did not know the answer”. As can be seen from Figure 3a, most of the students (67%) agreed (i.e., including strongly and somewhat) that they received nudges whenever they did not give their best effort although 50% of the students had selected “I did NOT give my best effort” at least once right after they received each nudge. As can be seen from Figure 3b, 55% of the students agreed (i.e., including strongly and somewhat) that they received nudges whenever they did not know the answer.

### 3.4. Metacognitive Measures

**Figure 4:**
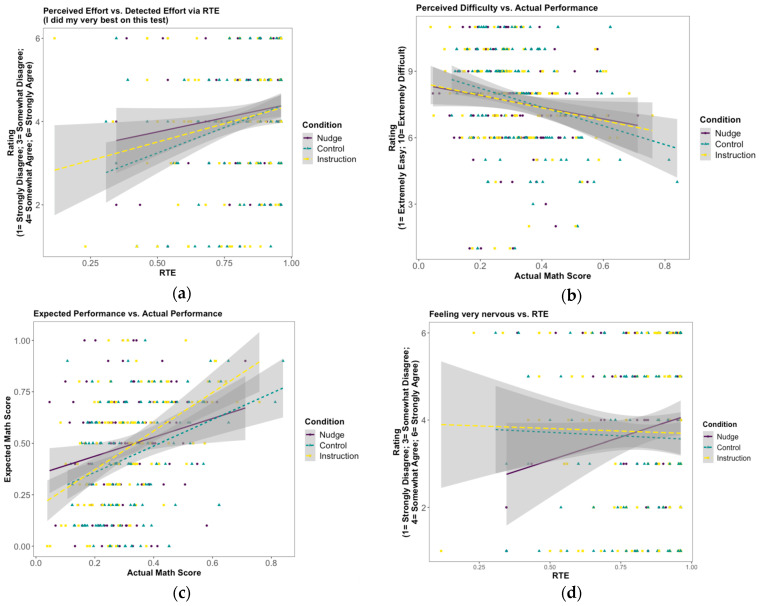
The relationship between (**a**) perceived effort and response time effort (RTE); (**b**) perceived difficulty and actual math score; (**c**) expected performance and actual performance; and (**d**) feeling very nervous due to the mathematics assessment and RTE in each condition.

**Figure A4:**
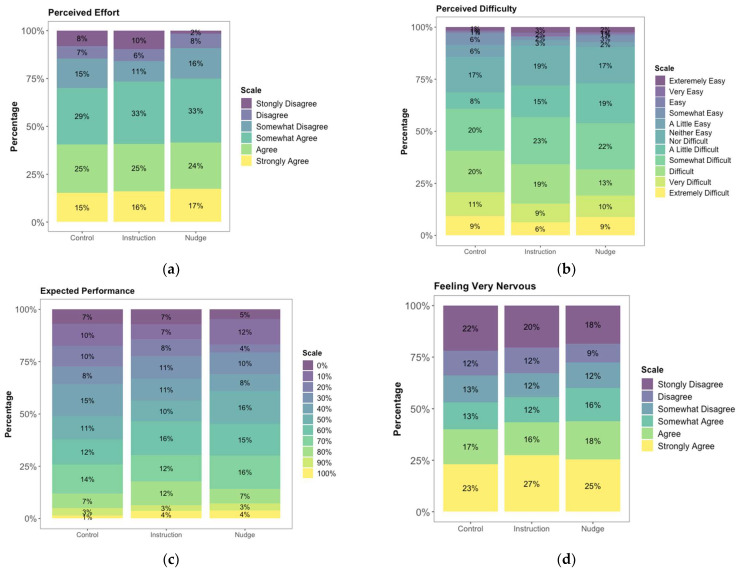
Percentages of each option in Likert Scale in each condition for (**a**) perceived effort, (**b**) perceived difficulty, (**c**) expected performance, and (**d**) feeling very nervous.

As we described in the Analysis subsection, we investigated the effects of experimental conditions on students’ perceived effort (“*I did my very best on this test*”), perceived difficulty (“*How difficult was the math assessment that you just completed?*”), expected performance (“*What percentage of questions do you think you answered correctly on the math assessment?*”), and feeling very nervous due to the activity (“*Working on this activity made me feel very nervous*”). Figure 4 shows the relationship between RTE or actual math score and observed ratings in each condition for each self-report measure (see Figure A4 in Appendix E for the percentages of each option in Likert Scale in each condition).

The results of the models showed that RTE had a positive significant association with the ratings for perceived effort and did not have a significant association with feeling “very nervous” as a result of taking the math assessment. The actual math score had a negative significant association with the ratings for perceived difficulty and had positive significant association with expected performance. The reason why we examined nervousness by RTE and whether this association differs by condition was related to the fact that students who had lower RTEs received more nudges in the nudge condition, which might have caused more nervousness compared to control condition. Although there were trends in the data, for all the above-mentioned self-report measures, there were no significant differences between nudge and control and effort instruction conditions (see Table A4 in Appendix E for the estimates, confidence intervals, and *p*-values of the models).

## 4. Discussion

The main goal of the present study was to investigate the effects of nudges on students’ cognitively disengaged behavior during a low-stakes mathematics assessment. To this end, we compared 8th-grade students’ response disengagement, performance (i.e., item score), as well as self-report metacognitive measures (i.e., perceived difficulty and effort, expected performance, and feeling very nervous) between nudge and control conditions, and nudge and effort instruction conditions. Below, we first discuss the theoretical implications of the findings. Subsequently, we briefly highlight the practical implications. Finally, we conclude by discussing the limitations of the present study together with future directions for research.

### 4.1. Theoretical Implications

*Effects of nudges on student effort, performance, and self-reported metacognitive measures*: As we mentioned in the introduction, cognitively disengaged behavior can be seen as a manifestation of System-1-driven behavior (quick, intuitive, and cognitively less demanding), and solution behavior can be seen as a manifestation of System 2 (Wise 2017). The fluctuation between System-1 and System-2-driven decision-making and behavior during assessment tasks (i.e., cognitive (dis)engagement) are affected by both internal (e.g., perceived effort, mental effort capacity, general cognitive abilities; Goldhammer et al. 2017; Lindner et al. 2019) and external parameters (e.g., item format, visual display of items; Wise et al. 2009; Lindner et al. 2019). Our main goal was to investigate whether it is possible to affect students’ cognitive (dis)engagement through nudges.

Similar to Wise et al.’s (2006) study, which was conducted with university students in the domains of scientific reasoning and fine arts, our findings indicate nudging students’ during assessment activity significantly decreased response disengagement (see Table 2) compared to control and effort instruction conditions by potentially shifting behavior from the undesirable, System-1-related cognitively disengaged behavior to a desirable, System-2-related solution one (see Wise 2017). The underlying shift from System 1 to System 2 might be related to students’ perception about the nudges, namely the feeling of “being monitored” or “being watched”, which might result in decreased response disengagement; thus, overriding their System-1-driven decision with the System-2-driven one through metacognitive processes. However, if the students had no initial motivation to go through the task, which might be due to several reasons (e.g., self-relevance, motivational framing; Finn 2015), we would not expect that nudges necessarily make students shift their behavior from cognitively disengaged to solution processes. Although we did not have a direct measure of student motivation, in our study, four students received a nudge on average 14 times (*SD* = 3) and on average students received nudges 4 times (*SD* = 4), which might indicate these four students were not motivated to complete the task and the nudges did not have an effect on their cognitive engagement. Note that we think that students’ mathematical knowledge and skills alone cannot explain these four students’ behavior because these students had both high and low school grades (i.e., two of them had grade D and F and one of them had an A, and we do not have information about the fourth student). The hypothesized effect of motivation on the impact of nudges on students’ test-taking behavior can be investigated with manipulation of the motivational framing of the study (i.e., increasing the motivation is expected to reduce the excessive number of nudges).

Furthermore, although the content of the nudge potentially had an impact on the magnitude of its effects, our findings indicate that the positive effect of nudges on student test-taking effort (i.e., cognitive engagement) cannot be solely attributed to its content. Although the effort instruction condition included a similar text with the nudge condition (but is only presented once before students started the assessment), students in the effort instruction condition had significantly higher cognitively disengaged responses compared to the control condition.

Although nudging students significantly improved their test-taking effort, it did not significantly affect performance after controlling for students’ mathematics class grades (see Table 2). However, similar to Wise et al. (2006), there was a positive shift in the scores in both Block 1 and Block 2 (see Figure 1). One implication of this pattern of results is that while nudges can significantly alter students’ cognitively disengaged behavior toward solution behavior, their scores may not improve correspondingly. The non-significant effects of nudging on performance could be attributed to students’ self-monitoring of their knowledge and required effort, opting for not-fully-effortful responses when they believed that they could not answer the question (e.g., Kolers and Palef 1977; Metcalfe and Finn 2008; Son and Metcalfe 2005). The previous literature on speeded feelings of knowing (i.e., a swift judgement of whether an answer could be retrieved from memory with some effort) suggests that people make initial evaluations of question prompts before attempting to answer, and subsequently use this information to select alternative strategies (Reder and Ritter 1992; Paynter et al. 2009). Moreover, these initial, rapid evaluations are found to be accurate in non-insight problem solving, such as with algebra problems (Metcalfe and Wiebe 1987).

Multiple lines of evidence from our study support the idea that students were self-monitoring their knowledge and required effort, and opting for not-fully-effortful responses when they knew that could not answer the question. First, we found that students with lower external math grades had higher item response disengagement (see Table 2; see also Goldhammer et al. 2017 and Lindner et al. 2019 for similar findings), which suggests that students with lower class grades knew when they did not know the answer. Second, students indicated that they had given their best effort after they received a nudge (see Figure 2). Students may have interpreted “giving their best effort” not only as exhibiting solution behavior (i.e., cognitively engaging with the problem solution) but also to an internal evaluation of their knowledge and skills, leading them to conclude that they did not have the knowledge to provide a correct response^2^. Third, 55% of the students agreed (i.e., including both strongly and somewhat agree) that they received nudges when they did not know the answer in the post-test (see Figure 3b).

In our investigation of the effects of the nudge condition on post-test self-reported measures (i.e., perceived difficulty and effort, expected performance, and feeling very nervous) compared to control and effort instruction conditions, we did not find any significant difference between conditions (although there were some trends; see Figure 4). However, there were significant positive relationships between: (a) response time effort (RTE) and perceived effort, and (b) students expected math test score and actual math score. As expected, there was a negative significant relationship between perceived difficulty and the actual math score. We did not find any relationship between RTE and feeling very nervous due to the math activity, which may indicate that nudges did not make students feel more nervous since lower RTE means that students would have received more nudges.

Note that, as we mentioned, a nudge was introduced when a response was both incorrect and below the predefined item time threshold in students’ first-attempt response. The rationale behind this decision was so that we would not frustrate students who may have given a correct answer by chance (in the multiple-choice/select items). Because nudges were given for incorrect responses but not correct responses that were below item time thresholds, students may have figured out that they received nudges only when their answers were wrong: thus, interpreting the nudge as feedback for their performance. However, we do not think that this possibility significantly affected our results since there was no significant difference between students’ expected performance between the nudge and control conditions (see Figure 4c).

*Motivation in low-stakes assessment:* Although students did not have to respond to any of the presented items (i.e., they could skip all items), and we did not provide any feedback to the students or teachers, the number of items that students responded to was high (~90%). This indicates that formal consequences about performance, receiving feedback about performance, and receiving direct incentives (i.e., we gave incentives to the schools but not directly to the students), or making tests intrinsically enjoyable or less taxing (Wise and DeMars 2005) are not the only driving forces for 8th-grade students’ response rate in an assessment context. Since the assessment task was a part of a class activity and teachers were present, we surmise that having teachers as proctors and taking the assessment task as a class activity might make 8th-grade students motivated enough to provide responses, although their responses were not always effortful (see also Lau et al. 2009 for the effects of proctor strategies on examinee effort).

### 4.2. Practical Implications

As we discussed in the introduction, detecting and reducing cognitively disengaged responses during assessment is particularly important for supporting the validity of the inferences about what students know and can do. Introducing item-based nudges during a computerized assessment task can be leveraged in low-stakes assessment contexts, such as in formative assessments, in classrooms, intelligent tutoring systems, and training modules for adults as well as in large-scale, low-stakes assessments such as NAEP or PISA to reduce cognitively disengaged responses during assessment.

In addition to education context, detecting cognitively disengaged responses and providing effort-based nudges can be also leveraged in online experiments with different populations such as adults or students for research purposes, where the goal is also to maximize effortful responding so that the interpretations of the experimental studies are more valid.

### 4.3. Limitations and Future Directions

There are a number of limitations of the current study that future research needs to investigate. First, to investigate our conclusion that some students may be giving not-fully-effortful responses strategically based on monitoring their knowledge and required effort, future studies should include a post-test question, or conduct an interview with students, in which students are asked to explain what it means for them to give their best effort. Alternatively, a third response option in the first nudge screen (see Figure A2 in Appendix B) can be added in which students are able to select “I knew that I didn’t know the answer” in addition to “I gave my best effort” and “I didn’t give my best effort” so that we have a data point for each nudge instance, which is expected to be a more accurate self-report measure.

Second, although we provided evidence against this possibility, it is still feasible that a small group of students perceived nudges as feedback for their performance since nudges only presented following “quick” and incorrect responses. To have more direct evidence, it is worthwhile to add Likert scale post-test questions asking students to rate “*I received warning messages whenever my response was incorrect*” together with other possible options (to prevent potential bias) “*I received warning messages whenever my response was correct*”, “*I received warning messages whenever my response was too quick*”, and “*I received warning messages whenever my response was too slow*”.

Third, although the nudges were based on the students’ responses (i.e., both “quick” and incorrect), it would be worthwhile to investigate further opportunities for nudges to adaptively influence behavior. For example, in our study, four students received a nudge frequently (i.e., on average 14 times). A remaining question is whether these students felt frustration when receiving the nudges. If yes, it may be wise to add a rule to the nudge algorithm such that nudges stop after a defined number of consecutive nudges. Another adjustment to investigate would be to stop nudges as a function of the remaining time left to finish the assessment task since students might show more cognitively disengaged behavior when there is little time left but there are still questions to be answered.

Another limitation of the current study concerns the identification of which specific aspects of the nudges were most effective in reducing cognitively disengaged responses. One possibility is that prompting students to reflect on whether they had given their best effort on a particular problem was the key factor, regardless of the timing of the nudges following a not-fully-effortful response. In the future, it may be valuable to introduce another condition in which students are asked multiple times, and at random intervals, to reflect on whether they are giving their best effort. Based on our findings, we hypothesize that targeted, timely nudges significantly decrease cognitively disengaged responses compared to random reflection prompts.

Finally, in our study, we chose to explicitly ask students to indicate whether they would go back to the item after receiving a nudge although it was possible to track students’ navigation between items from the log data. We made this decision because without an explicit indicator, it would be more difficult to make a valid inference about students’ purpose for revisiting an item (e.g., whether their goal was to go back to reevaluate their responses or simply to navigate between items without a reevaluation goal). Therefore, we could not conduct an analysis to investigate whether the rates of revisiting an item after a not-fully-effortful response significantly differs between the nudge condition and effort instruction and control conditions. Future research is needed to investigate whether students go back to their cognitively disengaged responses at a similar rate after receiving a nudge vs. without a nudge.

Overall, our findings suggest that nudges, but not effort instruction, significantly reduce cognitive disengagement during problem solving. However, most students appear to strategically modulate their level of effort based on self-monitoring their knowledge and estimated response effort.

## Figures and Tables

**Table 1:** A comparison of Wise et al. (2006) study and the present study.

Features	Wise et al. (2006)	The Present Study
Sample	University students	8th-grade students
Domain	Scientific reasoning and fine arts	Mathematics
Experimental manipulations	Warning vs. Control	Nudge vs. Control Nudge vs. Instruction
Disengagement measure	Rapid guessing behavior	Not-fully-effortful responses
Method to detect disengagement	Data-driven	Theory-driven
Item navigation	Students had to answer each item and they were not allowed to go back to an item after they submitted their answer.	Students could omit answers and they were able to navigate between items within a block.
Warning/Nudge algorithm	The first warning was presented after detecting three consecutive RGBs. The second warning was presented if the students had another three consecutive RGBs.	Students were presented with a nudge to give their best effort following each first-attempt response that was both incorrect and not-fully-effortful.
Outcome measures	Response time effort, total score	Item response disengagement, item score

**Table 2:** Odds ratios, confidence intervals (CI), and *p*-values of the final generalized mixed-effects model (binomial) for item response disengagement and item score.

	Item Response Disengagement			Item Score		
Predictors	Odds Ratios	CI (95%)	*p*-Value	Odds Ratios	CI (95%)	*p*-Value
(Intercept)	0.01	0.00–0.01	**<.001**	0.89	0.50–1.60	.700
Condition (Control)	1.44	1.06–1.96	**.021**	0.91	0.80–1.03	.144
Condition (Instruction)	1.60	1.17–2.18	**.003**	0.97	0.85–1.10	.584
Block (2)	2.87	2.55–3.24	**<.001**	0.28	0.24–0.34	**<.001**
Current MATH Grade (B)	2.62	1.81–3.79	**<.001**	0.88	0.82–0.94	**<.001**
Current MATH Grade (C)	4.13	2.81–6.05	**<.001**	0.6	0.52–0.69	**<.001**
Current MATH Grade (D)	5.48	3.58–8.37	**<.001**	0.38	0.33–0.45	**<.001**
Current MATH Grade (F)	8.09	5.26–12.43	**<.001**	0.32	0.27–0.39	**<.001**
Random Effects				Random Effects		
σ^2^	3.29			σ^2^	3.29	
τ_00 UserID:Teacher_	1.99			τ_00 UserID:Teacher_	0.28	
τ_00 ItemID_	0.63			τ_00 ItemID_	1.54	
τ_00 Teacher_	1.18			τ_00 Teacher_	0.29	
ICC	0.54			ICC	0.39	
N _UserID_	782			N _UserID_	780	
N _Teacher_	12			N _Teacher_	12	
N _ItemID_	26			N _ItemID_	26	
Observations	18,295			Observations	18,278	

## Data Availability

The data presented in this study are openly available in OSF at https://osf.io/4a68m/?view_only=116e28a7ce2f42e8b4909b68816b3c0c (accessed on 15 September 2023).

## Notes

1 The final models reported in the manuscript include all student responses to the items. To further investigate the effects of nudges, we also conducted two additional analyses which included a subset of data that excluded the students who did not receive or deserve any nudge (i.e., RTE = 1) and a subset of data that only included the data after students’ first detected not-fully-effortful response (i.e., after deserving first nudge). The results of both analyses showed similar patterns with the overall results that were reported in the manuscript although the effects of nudges were larger after excluding students who had RTE = 1 (i.e., RTE < 1). For brevity and simplicity, we included these additional analyses on disengaged responses and performance in Appendix D.

2 Alternatively, it might also be the case that students felt that they have found out and denied that they did not give their best effort, or alternatively they did not put effort to evaluate their effort and just clicked on the option that they gave their best effort (although, if it was the case, we would expect a more random selection of one of the presented options, possibly skewed by the last location of the mouse to decrease the effort to click on an option without effort).

## Appendix C

**Table A1:** Estimates, standard errors (SE), *z*-values, and *p*-values of the final generalized mixed-effects model (binomial) for item response disengagement and performance (i.e., item score).

	Item Response Disengagement				Item Score			
Predictors	Estimate	SE	*z*-Value	*p*-Value	Estimate	SE	*z*-Value	*p*-Value
(Intercept)	−5.097	0.403	−12,664	**<.001**	−0.15	0.298	−0.504	.614
Condition [Control]	0.364	0.158	2.305	**.021**	0.035	0.065	0.547	.584
Condition [Instruction]	0.470	0.158	2.981	**.003**	−0.058	0.065	−0.900	.368
Block [2]	1.056	0.061	17.463	**<.001**	−0.133	0.036	−3.703	**<.001**
Current Math Grade [B]	0.963	0.188	5.12	**<.001**	−0.515	0.073	−7.038	**<.001**
Current Math Grade [C]	1.417	0.195	7.257	**<.001**	−0.956	0.078	−12.239	**<.001**
Current Math Grade [D]	1.701	0.217	7.856	**<.001**	−1.128	0.09	−12.590	**<.001**
Current Math Grade [F]	2.090	0.220	9.523	**<.001**	−1.262	0.093	−13.553	**<.001**

## Appendix D

*Response disengagement and item score after excluding students who did not receive/deserve any nudges (i.e., RTE < 1)*: We conducted additional analyses by excluding students who did not receive any nudges in the nudge condition, and students who did not deserve any nudges in the effort instruction and control conditions.

Table A2 shows the estimated, standard errors (SE), *z*-values, and *p*-values of the final generalized mixed-effects model (binomial) for item response disengagement and item score after excluding students who had RTE = 1 (i.e., RTE < 1). The pattern of main results was similar to the overall results that were reported in the manuscript although the effects of nudges were larger after excluding students who had RTE < 1 (*cf* Table 2).

**Table A2:** Estimates, standard errors (SE), *z*-values, and *p*-values of the final generalized mixed-effects model (binomial) for item response disengagement and item score after excluding students who had RTE = 1 (i.e., RTE < 1).

	Item Response Disengagement for RTE < 1				Item Score for RTE < 1			
Predictors	Estimate	SE	*z*-Value	*p*-Value	Estimate	SE	*z*-Value	*p*-Value
(Intercept)	−3.531	0.291	−12.157	**<.001**	−0.531	0.290	−1.828	.068
Condition [Control]	0.383	0.134	2.862	**.004**	0.053	0.075	0.715	.475
Condition [Instruction]	0.590	0.135	4.368	**<.001**	0.027	0.076	0.350	.726
Block [2]	1.040	0.062	16.846	**<.001**	−0.182	0.048	−3.769	**<.001**
Current Math Grade [B]	0.384	0.170	2.264	**.024**	−0.367	0.091	−4.021	**<.001**
Current Math Grade [C]	0.659	0.173	3.807	**<.001**	−0.647	0.095	−6.836	**<.001**
Current Math Grade [D]	0.964	0.190	5.083	**<.001**	−0.951	0.107	−8.865	**<.001**
Current Math Grade [F]	1.109	0.187	5.942	**<.001**	−1.008	0.107	−9.451	**<.001**

*Response disengagement and item score after “deserving” first nudge (i.e., after first detected not-fully-effortful response)*: In the nudge condition, after deserving first nudge analysis included all the items that a student had responses after receiving first nudge. Similarly, in the instruction and control conditions, it included all the items that a student had responses after deserving first nudge.

Similar to overall RTE, students had higher RTEs after receiving/deserving first nudge in the nudge condition (*M* = 0.80, *SD* = 0.21) compared to the control condition (*M* = 0.72, *SD* = 0.22) and to the instruction condition (*M* = 0.72, *SD* = 0.24).

Table A3 shows the estimates, standard errors (SE), *z*-values, and *p*-values of the final generalized mixed-effects model (binomial) for item response disengagement and item score after deserving first nudge. The pattern of main results was similar to the overall results that were reported in the manuscript (*cf* Table 2).

**Table A3:** Estimates, standard errors (SE), *z*-values, and *p*-values of the final generalized mixed-effects model (binomial) for item response disengagement and performance after deserving first nudge.

	Item Response Disengagement after Deserving First Nudge				Item Score after Deserving First Nudge			
Predictors	Estimate	SE	*z*-Value	*p*-Value	Estimate	SE	*z*-Value	*p*-Value
(Intercept)	−6.212	0.597	−10.412	**<.001**	−0.080	0.308	−0.259	.068
Condition [Control]	0.756	0.262	2.883	**.004**	−0.010	0.075	−0.139	.890
Condition [Instruction]	0.820	0.261	3.138	**.002**	−0.146	0.075	−1.946	.052
Block [2]	0.629	0.093	6.785	**<.001**	−0.133	0.044	−3.006	**.003**
Current Math Grade [B]	1.292	0.319	4.056	**<.001**	−0.504	0.084	−5.988	**<.001**
Current Math Grade [C]	2.063	0.329	6.264	**<.001**	−1.003	0.090	−11.108	**<.001**
Current Math Grade [D]	2.538	0.360	7.052	**<.001**	−1.131	0.104	−10.878	**<.001**
Current Math Grade [F]	2.910	0.363	8.009	**<.001**	−1.270	0.108	−11.771	**<.001**

## Appendix E

**Table A4:** Estimates, confidence intervals (CI), and *p*-values of the final linear regression models for each self-reported measure.

	Perceived Effort			Perceived Difficulty			Expected Performance			Feeling Very Nervous		
Predictors	Estimates	CI (95%)	*p*-Value	Estimates	CI (95%)	*p*-Value	Odds Ratios	CI (95%)	*p*-Value	Estimates	CI (95%)	*p*-Value
(Intercept)	2.57	1.83–3.32	**<.001**	8.66	8.05–9.28	**<.001**	0.39	0.21–0.71	**.002**	3.56	2.52–4.60	**<.001**
Condition [Control]	−0.13	−0.44–0.18	.403	0.14	0.36–0.64	.587	0.79	0.49–1.26	.320	−0.21	−0.65–0.22	.339
Condition [Instruction]	−0.09	−0.43–0.25	.614	−0.00	−0.55–0.54	.989	0.96	0.57–1.62	.884	−0.08	−0.56–0.40	.744
RTE	1.94	1.12–2.76	**<.001**	-	-	-	-	-	-	0.32	−0.84–1.48	.587
Math Score	-	-	-	−3.39	−4.95–−1.82	**<.001**	17.03	3.75–82.00	**<.001**	-	-	-
Observations	416			Observations	397		Observations	401		Observations	410	
